# Oxidative Stress in Vascular Aging: Therapeutic Potential of the Antioxidant Paradox. A State-of-the-Art Review

**DOI:** 10.3390/antiox15091152

**Published:** 2026-09-10

**Authors:** Aleyma Veliz Perez, Mihaela Badea, Sehrish Bilal

**Affiliations:** 1 Department of Fundamental, Prophylactic, and Clinical Disciplines, Faculty of Medicine, Transilvania University of Brasov, 56 Nicolae Balcescu, 500019 Brasov, Romania; aleyma.veliz@unitbv.ro (A.V.P.); sehrish.bilal@unitbv.ro (S.B.); 2 Research Center for Fundamental Research and Prevention Strategies in Medicine, Research and Development Institute, Transilvania University of Brasov, 10 Institutului, 500484 Brasov, Romania

**Keywords:** oxidative stress, cardiovascular, antioxidants, aging, vascular health

## Abstract

Cardiovascular disease (CVD) remains the leading cause of mortality worldwide, with aging as one of its main risk factors. Cellular senescence and oxidative stress form a bidirectional vicious cycle that drives vascular aging, endothelial dysfunction, and atherosclerotic progression. This state-of-the-art review synthesizes current evidence on the interplay between oxidative stress and senescence in cardiovascular aging and critically assesses whether antioxidant strategies can benefit vascular health. A targeted literature search was conducted in PubMed/MEDLINE, Web of Science, and Scopus (2016–2026). While endogenous antioxidant defenses decline with age, exogenous antioxidants, including resveratrol, vitamins C and E, omega-3 fatty acids, carotenoids, and coenzyme Q10,demonstrate promising preclinical effects, yet large-scale clinical trials have yielded inconsistent results. The VITAL and STRENGTH trials failed to demonstrate significant cardiovascular benefit with omega-3 supplementation, and elevated serum β-carotene was found paradoxically associated with increased cardiovascular mortality. Emerging mitochondria-targeted antioxidants (MitoQ, MitoTEMPO) show preclinical promise but require further clinical validation. Current evidence does not support antioxidant supplementation for cardiovascular prevention. Lifestyle interventions, particularly antioxidant-rich dietary patterns such as the Mediterranean diet, remain the safest strategy. Future research should develop personalized approaches guided by oxidative stress biomarkers and long-term trials.

## 1. Introduction

Cardiovascular disease (CVD) continues to be the primary contributor to morbidity and mortality globally [1]. In the United States, national statistics indicate that coronary heart disease (CHD) accounted for approximately 39.5% of CVD-related deaths in 2022 [1]. Globally, an estimated 621 million individuals are affected by CVD, while approximately 18.6 million deaths are attributed to the disease each year [2,3]. In recent years, advances in science have significantly increased life expectancy, which has led to a rise in morbidity, especially age-related conditions [2].

Aging in humans is a well-established risk factor for a series of aging-related diseases, including cardiovascular disease, endothelial dysfunction, atherosclerosis, inflammation, and oxidative stress, which are all directly linked to the etiology and development of CAD [4,5,6].

Cellular senescence refers to a stress-induced, permanent cessation of the cell cycle that leads to adverse functional and structural changes [5]. Senescence impacts several aspects of the cardiovascular system, contributing to diseases such as atherosclerosis, myocardial infarction (MI), and heart failure (HF). Recently, the focus has been on understanding the processes involved in cardiovascular aging, including telomere shortening and damage, and oxidative stress [4].

Oxidative imbalance is characterized by the excessive production of reactive oxygen species (ROS); is essential for maintaining physiological homeostasis, while oxidative imbalance has been strongly implicated in the development and progression of numerous diseases, particularly those associated with ageing [7]. Elevated oxidative stress can disturb the physiological balance between vasodilatory and vasoconstrictive mechanisms by increasing the generation of reactive oxygen species (ROS) and vasoconstrictor mediators, including endothelin-1 (ET-1) and angiotensin II. These alterations contribute to an endothelium-dependent impairment of vasodilatory function within the microcirculation. Nitric oxide (NO) is thought to have a more prominent role in regulating epicardial coronary vessels, whereas endothelium-derived hyperpolarization (EDH)-mediated mechanisms are considered more important in the microvasculature. Consequently, the dysfunction of these respective pathways may contribute to endothelial impairment at the epicardial and microvascular levels. Oxidative stress is both a trigger and amplifier of vascular aging and atherosclerotic disease [7,8].

Effective interventions are needed to delay or attenuate age-related chronic diseases and promote a longer health span [8]. Targeting the oxidative stress pathway offers a promising therapeutic approach to mitigate these effects and slow CVD progression [9].

Antioxidant-based interventions are increasingly used in the management of age-associated diseases; however, a thorough understanding of how oxidative stress behaves under different physiological and pathological conditions remains essential [8]. Supplements like resveratrol, vitamins C and E, omega-3 fatty acids, flavonoids, and coenzyme Q10 have shown antioxidative properties and potential cardiovascular benefits [10].

This review explores the role of oxidative stress in CVDs and evaluates the current evidence in oxidative stress management in cardiovascular diseases by making a critical analysis of supplements like resveratrol, vitamins C and E, omega-3 fatty acids, flavonoids, and coenzyme Q10. Also, we emphasize the importance of precision and personalized medicine in CVD management, highlighting their importance for medicine strategies based on oxidative stress biomarkers.

## 2. Materials and Methods

### 2.1. Study Design

Given emerging evidence linking oxidative stress, senescence, and vascular aging, this manuscript is conducted as a state-of-the-art review to perform a critical, interpretive synthesis of current, high-quality evidence.

### 2.2. Literature Search Strategy

A literature search was performed in the following electronic databases: PubMed/MEDLINE, Web of Science, and Scopus. The literature research was conducted between April and June of 2026. The search was limited to articles published between 2016 and 2026. Search terms combined MeSH headings and free-text keywords using Boolean operators (“AND”, “OR”), and adapted to each database:

Core concepts: (“vascular aging” OR “endothelial dysfunction”) AND (“oxidative stress” OR “reactive oxygen species” OR “ROS”) AND (“cellular senescence” OR “senescence-associated secretory phenotype”).

Intervention concept: (“antioxidants” OR “vitamin C” OR “vitamin E” OR “beta-carotene” OR “polyphenols” OR “mitochondrial antioxidant”) AND (“cardiovascular” OR “atherosclerosis” OR “endothelial dysfunction”).

### 2.3. Inclusion Criteria

We included peer-reviewed articles published in English between 2016 and 31 March 2026. Regarding the study designs, original research, systematic reviews, meta-analyses, randomized controlled trials (RCTs), clinical trials, and mechanistic studies (in vivo or in vitro) were included. High-quality narrative reviews were also considered for contextual background. The article had to focus on at least two of these three concepts: oxidative stress, antioxidants, and vascular aging. Studies involving antioxidant interventions (dietary, supplemental, or pharmacological) were required to report at least one cardiovascular-relevant outcome. Studies published before 2015 (e.g., the HOPE, SELECT, and PHS-II trials) were considered exceptional as foundational evidence of antioxidant supplementation in cardiovascular aging.

### 2.4. Exclusion Criteria

Conference abstracts, editorials, opinion pieces, letters to the editor, preprints (not peer-reviewed), and case reports with fewer than 10 patients were excluded. Also, studies reporting non-cardiovascular outcomes without any vascular endpoint and observational studies with fewer than 100 participants.

### 2.5. Selection Process

All records retrieved from the electronic databases were exported to reference management software (Zotero 10.0.1). Duplicates were removed electronically and then manually verified. Titles and abstracts were screened independently by the authors against the inclusion and exclusion criteria. Full texts of potentially eligible articles were obtained and assessed in detail against the predefined criteria. Additionally, reference lists of included systematic reviews and meta-analyses were manually screened to identify any additional relevant studies not captured by the database search (snowballing).

### 2.6. Quality Assessment and Risk of Bias

To ensure adequate interpretation of the evidence presented, methodological quality was formally assessed using design-appropriate validated tools: AMSTAR 2 for systematic reviews and meta-analyses, RoB 2 for randomized controlled trials (RCTs), ROBINS-I for non-randomized intervention studies, the Newcastle–Ottawa Scale (NOS) for observational studies, and the SYRCLE risk-of-bias tool for preclinical animal studies. The assessment considered study design, sample size, methodological quality, risk of bias, and outcome characteristics. Detailed assessments are provided in Supplementary Table S1 [3,4].

Also, the following narrative quality considerations guided the interpretation of evidence:

Systematic reviews and meta-analyses: Preference was given to those with comprehensive search strategies, duplicate screening, and explicit risk-of-bias assessments (informally checked against AMSTAR-2 key items).

Randomized controlled trials (RCTs): Large, double-blind, placebo-controlled trials with adequate randomization, allocation concealment, low attrition, and intention-to-treat analysis were given greater weight.

Observational studies: Studies with larger sample sizes (≥100 participants), prospective designs, and adjustment for key confounders (age, sex, smoking, baseline antioxidant status) were prioritized.

Mechanistic studies: Preference was given to studies that used multiple lines of evidence (e.g., genetic and pharmacological interventions) and were published in peer-reviewed journals with an established reputation in redox biology or vascular aging.

Key methodological limitations of the included studies (e.g., short follow-up periods, inappropriate dosing, small sample sizes, and lack of baseline antioxidant measurements) are critically discussed in the narrative synthesis. No study was excluded solely based on quality.

### 2.7. Data Synthesis

A narrative, thematic synthesis was conducted following the following hierarchical structure. No meta-analysis was performed. The synthesis was organized into five main sections, reflecting the core question of whether antioxidants can delay cardiovascular aging through modulation of oxidative stress and senescence:

- Aging and Senescence in Cardiovascular Diseases
- Oxidative stress and cardiovascular diseases
- Oxidative Stress as an Inducer and Amplifier in Cardiovascular Diseases
- The Senescence -Oxidative Stress Axis
- Antioxidants Against Vascular Aging (Dietary Antioxidants, Mitochondrial-Targeted Antioxidants)
- The Antioxidant and Current Limitations: Personalized Cardiology
- Possible Antioxidant Interactions
- Senolytic Therapies: An Emerging Strategy for Vascular Aging

### 2.8. Limitations of the Review

Several limitations inherent to the state-of-the-art review methodology should be noted for the correct interpretation of this review. As a state-of-the-art review, selection was prioritized rather than exhaustive, which may have excluded relevant lower-impact studies. Only English-language articles were included. These limitations are mitigated by transparent reporting and critical discussion of conflicting evidence.

## 3. Results

### 3.1. Aging and Senescence in Cardiovascular Diseases

#### 3.1.1. The Aging Vasculature

Young vasculature is distinguished by intact and efficient endothelial function, characterized by nitric oxide (NO) synthesis for vascular homeostasis, inhibition of platelet aggregation, and prevention of leukocyte adhesion. These vessels are elastic and exhibit efficient endothelium-dependent relaxation, with adequate NO production, the most important vasodilator [11,12].

The aging vasculature undergoes progressive structural and functional deterioration, marked by compromised vasodilatory capacity due to diminished nitric oxide (NO) bioavailability and elevated expression of adhesion molecules, all of which contribute to endothelial dysfunction [11,12,13,14].

The disruption of the endothelial balance between vasodilators and vasoconstrictors impairs function and promotes pathological vascular remodeling. Aging is characterized by progressive structural and functional alterations in the arterial wall, especially in arterial intimal thickness and the extracellular matrix (ECM), which alters the structural and biomechanical properties of the arterial wall [11,12].

Collagen content increases markedly; in human aortic tissue, collagen concentration rises by up to 72% between ages 14 and 90 years, while elastin fibers become progressively disorganized, thinner, and fragmented. These changes are driven by increased matrix metalloproteinase (MMPs) activity, particularly MMP-2 and MMP-9, which degrade elastin and promote arterial fibrosis [11,12,13]. The resulting shift in the elastin/collagen ratio compromises arterial compliance, increases wall rigidity, and impairs the vessel’s ability to adapt to hemodynamic changes. Consequently, ECM stiffening promotes endothelial dysfunction by impairing shear stress-mediated NO production and by activating mechanosensitive signaling pathways that upregulate pro-inflammatory and pro-fibrotic gene expression in endothelial cells and vascular smooth muscle cells (VSMCs) [12]. VSMCs also undergo phenotypic switching from a contractile to a synthetic and pro-inflammatory phenotype in response to ECM stiffening, further contributing to vascular remodeling and atherosclerotic progression [8,13].

Furthermore, changes in the mechanical microenvironment can affect vascular smooth muscle cells and endothelial cells, contributing to endothelial dysfunction, inflammation, and further vascular remodeling. ECM remodeling and increased arterial stiffness represent important structural components of vascular aging that interact with oxidative stress and cellular dysfunction to promote progressive vascular deterioration. The resulting arterial stiffening is not merely a passive consequence of aging but an active, modifiable process that precedes and predicts cardiovascular events [11]. Ageing is associated with greater susceptibility to oxidative imbalance, largely as a result of declining antioxidant defenses and increased oxidative activity. This disruption in redox homeostasis has been implicated in the development of numerous pathological conditions, with a particularly strong association observed in diseases related to ageing [14].

#### 3.1.2. Cellular Senescence

**Figure 1:**
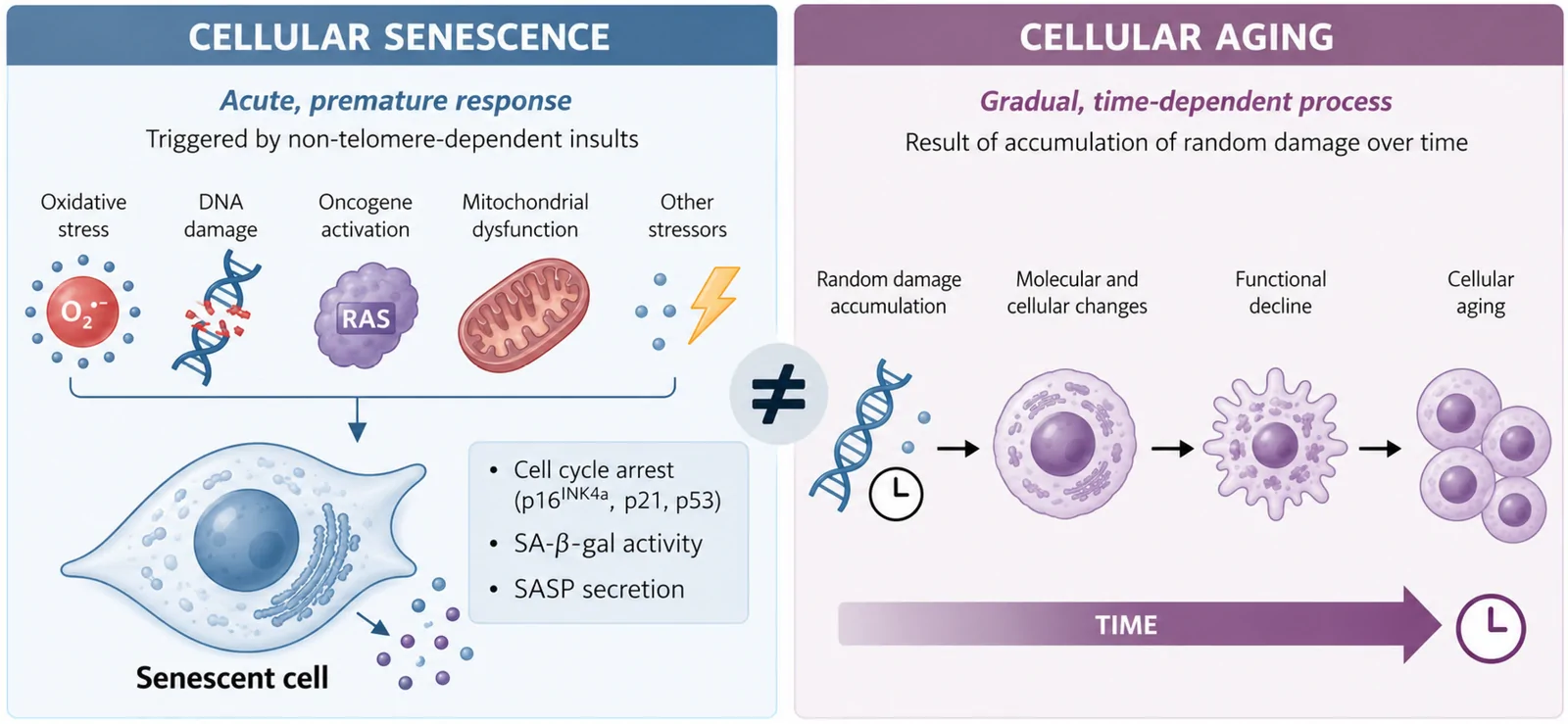
Differences between cellular senescence and aging. Schematic comparison of cellular senescence (**left panel**) and cellular aging (**right panel**). Vertical arrows (senescence) indicate cause–effect relationships: each stressor leads to a specific senescent response (cell-cycle arrest, SA-β-gal, SASP). Horizontal arrows (aging) represent the progressive and cumulative accumulation of damage and functional decline over time. The “≠” symbol highlights that senescence and aging are related but biologically distinct processes.

It is important to mention that the terms “senescent” and “aged” are not the same and should not be used interchangeably. Cellular senescence may arise prematurely in response to various stressors that are independent of telomere shortening, occurring before cells reach the limits of their replicative capacity, a process commonly referred to as stress-induced senescence [8,14]. In contrast, cellular ageing is characterized by the gradual accumulation of intrinsic cellular alterations resulting from persistent, low-level damage over time. These progressive changes ultimately contribute to impaired cellular function [8,14,15]. In short, not all senescent cells are aged cells [14], as is represented in Figure 1.

In Figure 1, the main differences between senescence and aging are represented. It is still very important to mention that cellular senescence represents a hallmark of aging, and senescent cells become more abundant in aged tissues.

With aging, cells progressively lose their proliferative capacity because of telomere attrition (replicative senescence), consequently becoming unable to replace damaged tissues and contributing to organismal dysfunction over time [14].

Cellular senescence is defined as a state of cell-cycle arrest triggered by various stressors, with permanent cessation of the cell cycle, resulting in adverse functional and structural changes [11,14]. A hallmark of senescence is the acquired senescence-associated secretory phenotype (SASP), characterized by the release of a range of bioactive molecules that can influence the surrounding microenvironment [14,15].

Cells of any age can undergo senescence. Actually, it occurs throughout our life; in developing and young organisms, cellular senescence prevents the propagation of damaged cells and contributes to tissue formation and homeostasis, whereas in old organisms, senescent cells start to accumulate because either the rate of their formation is increased or a deregulated immune system fails to remove them [14,15].

In the vasculature, cellular senescence primarily affects endothelial cells (ECs) and vascular smooth muscle cells (VSMCs), which are essential for maintaining vascular homeostasis and arterial structure [5,14]. Senescence can be induced by persistent stressors, including oxidative stress, DNA damage, telomere dysfunction, chronic inflammation, and altered mechanical stimuli [5,14]. At the molecular level, these stress signals activate the p53/p21 and p16^INK4a^/retinoblastoma (Rb) pathways, resulting in sustained cell-cycle arrest and the acquisition of the senescence-associated secretory phenotype (SASP), characterized by the secretion of pro-inflammatory cytokines, chemokines, and matrix-remodeling factors [5,14,15].

Senescent ECs have reduced vasodilatory capacity (reduced NO bioavailability), increased adhesion molecule expression, and a pro-thrombotic phenotype, while senescent VSMCs contribute to arterial stiffness through excessive matrix metalloproteinase (MMP) secretion and phenotypic switching toward a synthetic, pro-inflammatory state [11,14]. Significantly, senescence establishes self-perpetuating feed-forward loops: SASP factors from senescent cells induce paracrine senescence in neighboring cells, amplify local inflammation, and promote further oxidative stress, which in turn accelerates additional senescence and vascular damage [11,14].

The localized accumulation of senescent cells at sites of vascular injury amplifies tissue remodeling and contributes to plaque instability, fostering a pro-thrombotic, pro-fibrotic microenvironment. Senescent cell accumulation in the intimal and medial layers is implicated in both initiation and progression of atherosclerotic plaques [11,14].

Collectively, the accumulation of senescent cells in the vasculature promotes chronic vascular inflammation, endothelial dysfunction, extracellular matrix remodeling, and arterial stiffness, thereby accelerating vascular aging and increasing cardiovascular disease risk [5,11,14].

Importantly, senescent cell burden and SASP have been proposed as modifiable therapeutic targets rather than purely descriptive markers of vascular age, with senolytic and xenogeneic agents currently being explored to ameliorate age-related vascular dysfunction [14].

### 3.2. Oxidative Stress as an Inducer and Amplifier in Cardiovascular Diseases

Oxidative stress occurs when the equilibrium between pro-oxidant and antioxidant systems is disrupted, leading to the overaccumulation of free radicals and non-radical reactive oxygen species (ROS), such as superoxide (O_2_^•−^), hydrogen peroxide (H_2_O_2_), hydroxyl radical (^•^OH), ozone, and singlet oxygen (^1^O_2_). These species are generated through cellular mechanisms and external sources [15,16].

In the cardiovascular system, ROS originate from multiple sources, including the mitochondrial electron transport chain, NADPH oxidases (NOX), xanthine oxidase, and uncoupled endothelial nitric oxide synthase (eNOS). The imbalance between ROS production and the body’s antioxidant defense mechanisms, often seen in conditions like atherosclerosis and hypertension, promotes oxidative stress. Oxidative stress is implicated in the activation of signaling pathways associated with cardiac remodeling, hypertrophy, and apoptosis, exacerbating cardiovascular disease (CVD) progression [17,18,19].

The earliest pathophysiological stage in cardiovascular disease is endothelial dysfunction, a key antecedent to atherosclerosis, hypertension, and heart failure [19]. Oxidative stress has been proven to contribute to endothelial dysfunction by promoting lipid peroxidation and initiating atherosclerotic progression [11,13]. A key product of this lipid peroxidation is oxidized low-density lipoprotein (oxLDL), which plays a pivotal role in vascular aging by triggering inflammation and oxidative stress through its interaction with the lectin-like oxidized LDL receptor-1 (LOX-1) on endothelial cells. Binding oxLDL to LOX-1 increases the generation of reactive oxygen species, which in turn induces further LOX-1 expression and additional LDL oxidation, establishing a self-amplifying redox cycle. This redox-inflammatory amplification can impair endothelial function, reduce endothelium-dependent vasodilation, and promote vascular inflammation and atherogenic remodeling [8,11,13]. Increased oxidative stress may damage the endothelium, impair endothelium-dependent vascular relaxation, and increase vascular contractile activity. This imbalance activates the immune system and further promotes the release of cytokines and chemokines, which promote oxidative stress and ROS production [11,17].

A central mechanism by which oxidative stress amplifies vascular damage is through the redox-sensitive activation of the transcription factor nuclear factor kappa B (NF-κB). Elevated ROS directly activate NF-κB signaling, driving overexpression of pro-inflammatory cytokines (TNF-α, IL-1β, IL-6) and endothelial adhesion molecules, including intercellular adhesion molecule-1 (ICAM-1) and vascular cell adhesion molecule-1 (VCAM-1) in endothelial cells. This leads to increased leukocyte adhesion and infiltration into the vessel wall, promoting chronic inflammation and further ROS production. This oxidative stress–NF-κB–adhesion molecule axis increases monocyte adhesion and transendothelial migration, a rate-limiting early step in atherogenesis. This oxidative stress–NF-κB–adhesion molecule axis provides an important mechanistic link between redox imbalance, endothelial dysfunction, and vascular aging [11,12,19].

The resulting feed-forward cycle between oxidative stress and inflammation is an important feature of vascular aging and contributes to the progression of atherosclerosis and endothelial dysfunction [11,20]. In parallel, dysregulated angiotensin II (AngII) signaling is elevated with age; it promotes both NOX-dependent and mitochondrial ROS production, with mitochondrial ROS that can stimulate NADPH oxidase activity, creating an additional vicious cycle [20]. This amplification may be further exacerbated by endoplasmic reticulum (ER) stress, which can increase ROS generation and promote calcium-dependent mitochondrial dysfunction, thereby contributing to additional ROS generation [20].

Collectively, these interconnected mechanisms establish a self-perpetuating cycle of oxidative stress, inflammation, and cellular dysfunction that accelerates vascular aging and drives the progression of cardiovascular disease [11,19,20].

More than a simple initiator, oxidative stress is also an amplifier of vascular injury. Increased ROS production sets a cascade of self-sustaining pathways that perpetuate and intensify vascular damage [18,19].

### 3.3. The Senescence–Oxidative Stress Axis

**Figure 2:**
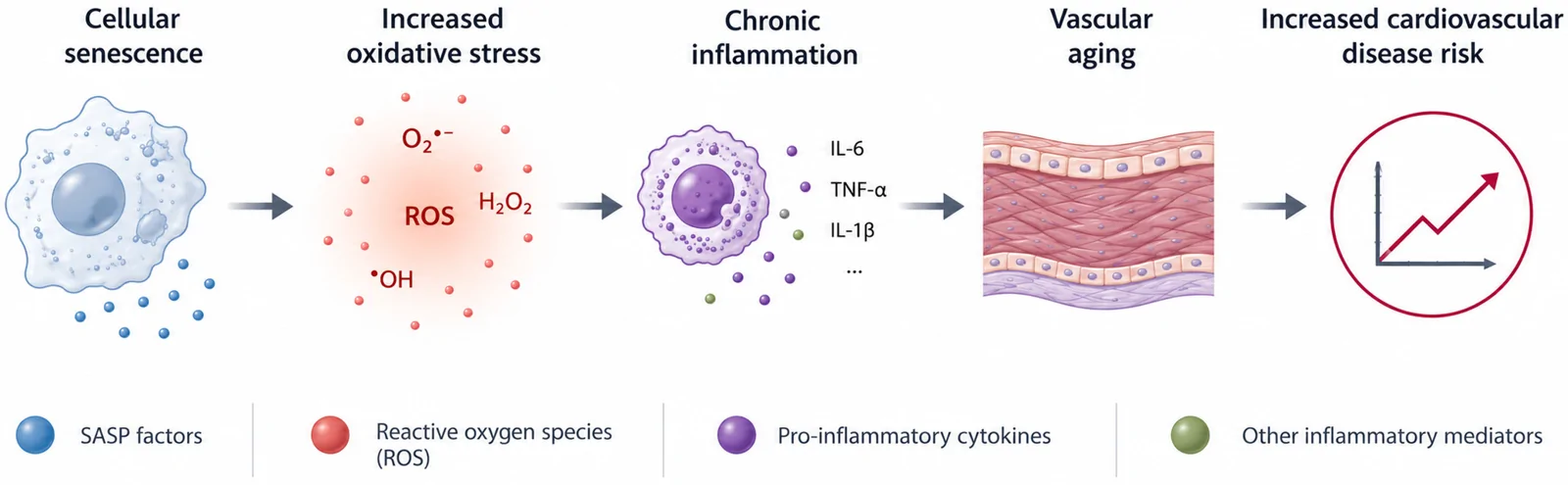
The senescence–oxidative stress axis. This figure illustrates the bidirectional relationship between cellular senescence and oxidative stress. Senescent cells promote ROS accumulation (superoxide, hydrogen peroxide, hydroxyl radicals), which in turn amplifies chronic inflammatory signaling via pro-inflammatory mediators (IL-6, TNF-α, IL-1β). This self-reinforcing senescence–oxidative stress–inflammation axis drives increased cardiovascular risk.

Cellular senescence contributes to oxidative stress by suppressing telomerase activity, while oxidative stress itself is a recognized trigger for cellular senescence [11]. This reciprocal relationship forms the basis of a vicious cycle [11,14,15] (Figure 2).

Recognizing oxidative stress as a consequence and an inducer of cellular senescence has motivated therapeutic strategies designed to mitigate oxidative damage, which have shown potential in delaying senescence-related phenotypes [14].

Chen et al. investigated the molecular mechanisms linking Klotho (an aging suppressor gene) deficiency to cardiac aging. This preclinical study used Klotho-deficient mice, aged mouse hearts, and cultured cardiomyocytes. Their results demonstrated that loss of Klotho markedly impaired the nuclear factor erythroid 2–related factor 2 (Nrf2)–glucocorticoid receptor (GR) signaling pathway, leading to reduced transcription of antioxidant genes, increased reactive oxygen species (ROS) accumulation, mitochondrial dysfunction, and oxidative DNA damage. These alterations promoted cellular senescence, as evidenced by increased expression of p16^INK4a^, p21, and senescence-associated β-galactosidase activity, resulting in cardiac hypertrophy, fibrosis, and functional deterioration. Notably, restoration of Nrf2 signaling decreased oxidative stress and reduced the senescent phenotype [16].

This study showed how restoration of Nrf2 activity attenuates oxidative damage and prevents cardiac remodeling, highlighting oxidative stress as both a trigger and amplifier of senescence.

### 3.4. Antioxidants Against Vascular Aging

Antioxidants are essential regulators of redox balance and cellular homeostasis, playing a central role in preventing oxidative stress. They preserve cellular redox homeostasis by delaying, preventing, or inhibiting oxidative reactions through the neutralization of reactive oxygen and nitrogen species (ROS/RNS) and the interruption of oxidative chain reactions [20,21].

Antioxidants can be organized into endogenous and exogenous antioxidants. Endogenous antioxidants are intrinsic defense mechanisms synthesized by the body to stabilize the continuous generation of reactive oxygen and nitrogen species (ROS/RNS). They include both enzymatic and non-enzymatic components that cooperate to preserve redox homeostasis and protect cellular macromolecules from oxidative injury. Exogenous antioxidants are compounds obtained primarily through diet or supplementation that complement endogenous antioxidant defenses [21,22]. Table 1 summarizes the main endogenous and exogenous antioxidants.

Several antioxidant compounds have been reported to alleviate oxidative stress and contribute to blood pressure regulation by neutralizing reactive species, thereby limiting free-radical chain reactions and supporting the maintenance of normal endothelial function [17]. Importantly, exogenous compounds investigated for their potential effects on oxidative stress should not be considered a biologically homogeneous class. Their chemical properties, cellular localization, pharmacokinetic profiles, bioavailability, and primary molecular targets differ substantially, which may influence both their biological effects and their clinical efficacy. Accordingly, clinical findings obtained with one compound should not be assumed to be transferable to other agents.

#### 3.4.1. Dietary Antioxidants

Epidemiological evidence consistently supports that diets rich in fruits, vegetables, olive oil, tea, and red wine, characteristic of the Mediterranean dietary pattern, are associated with reduced cardiovascular risk and attenuation of vascular aging [22,23].

In the CORDIOPREV (Coronary Diet Intervention with Olive Oil and Cardiovascular Prevention) clinical trial, Rivas Garcia et al. investigated the association between the overall antioxidant capacity of the diet and changes in common carotid artery intima-media thickness (IMT-CC) following a five-year dietary intervention based on two healthy eating patterns: the Mediterranean diet and a low-fat diet [24].

They collected 805 patients with coronary heart disease who followed a Mediterranean diet supplemented with ≥40 mL/day of olive oil, and long-term follow-up demonstrated significantly reduced carotid intima-media thickness (CIMT) progression, evaluated by echocardiography, compared to those on a low- fat control diet [24].

If we consider that CIMT is a sensitive and non- invasive marker in the early detection of atherosclerosis and in the prediction of future cardiovascular events [23]. These findings suggest that sustained consumption of antioxidant-rich dietary patterns, especially those enriched with extra virgin olive oil, may preserve vascular structure and delay the progression of subclinical atherosclerosis [24].

Also, the PREDIMED trial results demonstrated the relation of the Mediterranean diet with lower CVD incidence. This trial measured 62 phenolic metabolites in spot urine by liquid chromatography–high-resolution mass spectrometry at baseline and after 1 year in 1180 individuals: 653 incident CVD cases (stroke, myocardial infarction, CVD death, or heart failure) and a random subcohort of 603 participants (76 overlapping cases). This trial identified a urinary multi-metabolite signature of MedDiet adherence that was prospectively associated with a lower incidence of CVD [25].

Evidence from long-term, multicenter randomized controlled trials indicates that adherence to a Mediterranean-style dietary pattern may help limit the progression of subclinical atherosclerosis, potentially through its anti-inflammatory and antioxidant effects.

##### Resveratrol

Resveratrol (3,5,4-trihydroxystilbene) is a naturally occurring non-flavonoid polyphenolic compound belonging to the stilbene family, synthesized by plants as a phytoalexin in response to environmental stress, pathogen infection, ultraviolet radiation, and mechanical injury. It is mostly found in grapes, red wine, berries, and peanuts. Its low aqueous solubility and extensive first-pass metabolism result in poor oral bioavailability (<1%), with rapid glucuronidation and sulfation in the intestine and liver [26].

Due to its pleiotropic biological activities, resveratrol has attracted attention as a natural bioactive compound with antioxidant, anti-inflammatory, cardioprotective, neuroprotective, and anti-aging properties [26].

Pollack et al. conducted a randomized, double-blind, placebo-controlled crossover trial to evaluate the effects of resveratrol supplementation on vascular function, mitochondrial biology, and glucose metabolism in older adults with impaired glucose tolerance [27]. Participants (*n*:30) received high-dose resveratrol (2–3 g/day) or placebo for six weeks before crossing over to the alternate treatment. Resveratrol significantly improved endothelial function, as demonstrated by an increase in the Reactive Hyperemia Index (RHI), and increased mitochondrial number in skeletal muscle, accompanied by transcriptional changes enriched in mitochondrial function and oxidative phosphorylation pathways [28].

Marques et al. also investigated the effects of resveratrol acute supplementation; they collected 24 patients between 45 and 65 years old with hypertension and baseline endothelial dysfunction and conducted a randomized, cross-over, double-blind, placebo-controlled trial with trans-resveratrol supplementation. Blood pressure (BP) measurements, aortic systolic blood pressure (SBP), and brachial flow-mediated dilation (FMD) were performed. They found that FMD was significantly increased in women (4.2 ± 0.5 vs. 7.1 ± 1.3%, *p* = 0.026) but not in men (4.4 ± 0.9 vs. 4.9 ± 0.8%, *p* = 0.588) in the trans-resveratrol group. No changes in blood pressure were found. improves endothelial function in treated hypertensive patients with endothelial dysfunction [27].

These findings suggest that supplementation with resveratrol may promote an improvement in endothelial function, although the samples of both studies were too small.

##### Carotenoids

Carotenoids are a family of over 600 lipid-soluble C40 tetraterpenoid pigments, with β-carotene being the most abundant in human tissues; it is not an essential nutrient, and it is synthesized by plants, bacteria, and fungi. As lipid-soluble compounds, their absorption depends on the presence of dietary fats and is influenced by individual variations in intestinal uptake and conversion to retinol [29].

β-carotene is also the most potent provitamin A carotenoid. It serves as a direct precursor of retinol and acts as a direct quencher of singlet oxygen and a scavenger of peroxyl radicals. Its antioxidant potential has generated increasing attention. Increasing evidence has analyzed the effect of carotenoids in the prevention and treatment of age-related diseases [29,30].

A Japanese in vitro study in cultured vascular endothelial cells demonstrated that natural β-carotene suppressed oxidative injury to cultured vascular endothelial cells induced by hydrogen peroxide. The results of this mechanistic evidence show that oxidative stress induced by hydrogen peroxide decreased cell viability, and the addition of natural β-carotene significantly suppressed this oxidative injury, especially in the early stages of relatively weak oxidative stress [31].

Qiu, Z et al. investigated the associations of serum carotenoids with the risk of cardiovascular mortality among individuals with type 2 Diabetes. They included 3107 individuals with type 2 diabetes (T2D) from NHANES III and NHANES 2001–2006 [32]. Serum concentrations of α-carotene, β-carotene, β-cryptoxanthin, lycopene, and lutein/zeaxanthin were measured at baseline, and participants were followed for approximately 14 years. In this study, we found that higher concentrations of serum β-carotene were significantly associated with an increased risk of cardiovascular mortality, contrary to its expected antioxidant benefits (HR 2.47, 95% CI 1.62–3.76; *p* for trend = 0.002).

The association followed a clear dose–response pattern, meaning the risk of cardiovascular death rose steadily with higher serum β-carotene levels. A one-unit increment in natural log-transformed serum β-carotene was associated with a 46% higher risk of cardiovascular mortality (*p* = 0.001) [32]. These results break the traditional concept that higher antioxidant levels invariably confer cardiovascular protection. Antioxidants should not be considered a homogeneous group of compounds with universally beneficial effects [32].

##### Omega-3

Marine-derived long-chain n-3 fatty acids (eicosapentaenoic acid, EPA; docosahexaenoic acid, DHA) are polyunsaturated fatty acids that are highly lipophilic and incorporate into cell membranes, where they influence membrane fluidity, eicosanoid production, and inflammatory signaling. Their effects on antioxidant defenses are through complex mechanisms, including modulation of lipid mediators and membrane remodeling. They may be one of the most popular antioxidants. Evidence from animal research, small randomized controlled trials, and observational epidemiological studies suggests that these interventions may be beneficial in the primary prevention of cardiovascular disease. But in midsize-to-large trials, the evidence is still inconsistent [33].

One of the largest studies about this antioxidant is the VITAL (NEJM, 2019) trial [34]. This trial evaluated the effects of omega-3 supplementation (1 g/day) in 25,900 patients with no previous cardiovascular disease and a median follow-up of 5.3 years. The primary composite endpoint: major cardiovascular events (myocardial infarction, stroke, and death from cardiovascular causes) did not reach statistical significance (386 events in the n-3 group vs. 419 in the placebo group; HR 0.92, 95% CI 0.80–1.06; *p* = 0.24). Also, no significant effect was observed for total stroke (HR 1.04, 95% CI 0.83–1.31), death from cardiovascular causes (HR 0.96, 95% CI 0.76–1.21), or coronary artery bypass grafting (HR 0.99, 95% CI 0.73–1.33) [34].

The STRENGTH study (JAMA, 2020) evaluated the benefits of omega-3 supplementation in more than 13,000 patients with cardiovascular risk. They compared high-dose omega-3 fatty acids (4 g/day EPA + DHA) with a corn oil placebo [35].

This large multicentric trial demonstrated that high-dose omega-3 fatty acid supplementation did not reduce major adverse cardiovascular events compared with placebo in high-risk patients, despite significant increases in circulating omega-3 levels.

The trial was even terminated early for futility because it was unlikely to demonstrate any cardiovascular benefit. Also, the omega-3 group exhibited a higher incidence of atrial fibrillation compared with the placebo group [35].

These clinical trials showed no cardiovascular benefit with omega-3 supplementation. Vascular damage accumulates progressively over time and becomes increasingly difficult to reverse once structural remodeling has occurred. Although the STRENGTH trial was conducted in patients with cardiovascular risk, the VITAL trial was centered on patients with no cardiovascular risk. It should also be noted that the effectiveness of antioxidant therapies is influenced by multiple factors, including baseline oxidative stress, disease severity, dosage, treatment duration, and bioavailability [11,15].

##### Curcumin

Curcumin is a polyphenolic compound derived from the rhizome of turmeric (Curcuma longa), a spice commonly used in Asian cuisine and traditional medicine. In preclinical studies, dietary curcumin has demonstrated anti-inflammatory and antioxidant properties through the inhibition of NF-κB and MAPK signaling pathways, downregulation of COX-2 and iNOS, and reduction in pro-inflammatory cytokines such as TNF-α and IL-6. However, the translation of these dietary benefits to isolated curcumin supplementation has been limited by its poor oral bioavailability, rapid metabolism, and extensive first-pass effect [21,23].

In a primary experimental study, Panthiya et al. investigated hexahydrocurcumin (HHC), a major metabolite of curcumin, in angiotensin II (AngII) stimulated rat aortic vascular smooth muscle cells (VSMCs). Treatment with Ang II (10 µM, 24 h) significantly induced VSMC proliferation (*p* < 0.001), migration, and inflammatory responses. HHC (20 µM, 24 h) significantly attenuated these effects by modulating key molecular pathways: it inhibited Ang II-induced cyclin D1 increase and p21 decrease; reduced reactive oxygen species generation; and suppressed the expression of pro-inflammatory and pro-remodeling mediators, including nuclear NF-κB p65 (~0.5-fold decrease), TNF-α (~0.4-fold decrease), IL-6 (~0.3-fold decrease), and MMP-9 (~0.2-fold decrease) compared to Ang II-treated controls [36].

Despite the promising preclinical evidence for curcumin, its clinical application as an isolated supplement has been constrained by significant pharmacokinetic limitations. To overcome these limitations, advanced formulations have been developed, including nanodispersed and liposomal curcumin [36,37].

Santos-Parker et al. developed a randomized, placebo-controlled intervention that included 39 healthy adults, aged 45–74 years (curcumin group: n = 20, placebo: n = 19) to evaluate the effects of curcumin supplementation for 12 weeks. Participants were healthy men and postmenopausal women. They used Longvida^®^ curcumin, 2000 mg/day. The study demonstrated that curcumin significantly improved brachial artery flow-mediated dilation (FMD), a gold-standard measure of endothelial function, from ~6.0% to ~7.7% (*p* < 0.01), while no change was observed in the placebo group. Mechanistically, curcumin increased basal NO-mediated FMD by ~2.5% (*p* < 0.01) and elevated plasma nitrate/nitrite levels by ~8 µM (*p* < 0.05), indicating enhanced nitric oxide (NO) bioavailability. However, curcumin did not significantly modify large-artery stiffness or circulating biomarkers of oxidative stress and inflammation. These findings suggest a beneficial effect on endothelial function in middle-aged and older adults, but do not demonstrate reversal of vascular aging or systemic oxidative stress [37].

In addition, Dastani et al. performed a randomized, double-blind, placebo-controlled clinical trial to evaluate the effects of nano-curcumin (80 mg/day for 90 days) in 64 patients with type 2 diabetes mellitus and mild-to-moderate coronary artery disease (<70% stenosis confirmed by angiography). The study demonstrated that nano-curcumin significantly reduced high-sensitivity C-reactive protein (hs-CRP) levels (*p* < 0.001) and lipoprotein(a) (Lp(a)) levels (*p* = 0.043) compared to placebo. The mean percentage change (%Δ) in hs-CRP and Lp(a) was also significantly reduced in the nano-curcumin group (*p* < 0.001 and *p* = 0.007, respectively). Both Lp(a) and hs-CRP are cardiovascular biomarkers; however, the study did not assess endothelial function, vascular cellular senescence, or longitudinal measures of vascular aging [38].

#### 3.4.2. Trace Elements

##### Zinc

Unlike polyphenolic antioxidants, selenium and zinc are minerals that contribute to redox homeostasis primarily through their roles as essential micronutrients involved in antioxidant enzymes and cellular defense mechanisms. Their biological effects are closely related to the maintenance of endogenous antioxidant capacity rather than to direct and indiscriminate scavenging of reactive species [20,21].

Zinc is an essential trace mineral that serves as a structural and catalytic cofactor for over 300 enzymes, including the antioxidant enzyme superoxide dismutase (SOD), which catalyzes the dismutation of superoxide radicals into hydrogen peroxide and molecular oxygen. Zinc also plays a role in maintaining endothelial integrity by modulating inflammatory responses and reducing oxidative stress through the inhibition of NADPH oxidase activity and the induction of metallothioneins. Epidemiological studies have suggested that adequate zinc status is associated with lower cardiovascular risk [20,21].

A GRADE-assessed systematic review and dose–response meta-analysis of randomized clinical trials by Nazari et al. [39] examined the effect of zinc supplementation on cardiovascular disease risk factors. The authors reported significant reductions in fasting blood glucose, insulin resistance, triglycerides, total cholesterol, and LDL cholesterol, alongside improvements in HDL cholesterol levels. However, effects on blood pressure and inflammatory markers were heterogeneous across trials and did not reach consistent significance. These findings suggest a modest but relevant role for zinc supplementation in modulating cardiometabolic risk factors, although does not yet support its use as an established cardiovascular preventive strategy [39].

Furthermore, Hamedifard et al. evaluated 12 weeks of combined magnesium and zinc supplementation in a randomized, double-blind, placebo-controlled trial with 60 patients with type 2 diabetes mellitus and coronary heart disease; randomized to placebo (*n* = 30) or intervention (*n* = 30). Participants received either 250 mg/day of magnesium oxide plus 150 mg/day of zinc sulfate (equivalent to 30 mg of elemental zinc) or placebo. In patients with T2DM and CHD, combined magnesium and zinc supplementation for 12 weeks increased total antioxidant capacity and total nitrite and reduced CRP compared with placebo. the combined supplementation group experienced a significant reduction in C-reactive protein (β = −0.85 mg/L, 95% CI −1.26 to −0.45; *p* < 0.001) and increases in total antioxidant capacity (β = 43.44 mmol/L, 95% CI 3.39–83.50; *p* = 0.03) and total nitrite (β = 5.13 μmol/L, 95% CI 1.85–8.41; *p* = 0.003). However, this is a study of a combination therapy (magnesium and zinc together), and the reported benefits cannot be attributed to zinc (or magnesium) alone [40].

##### Selenium

Selenium is a trace mineral and a critical component of selenoproteins, including glutathione peroxidases (GPx) and thioredoxin reductases, which catalyze the reduction of hydrogen peroxide and lipid hydroperoxides, thereby protecting cells from oxidative damage. Selenium deficiency has been associated with increased oxidative stress and cardiovascular disease risk in observational studies. The complexity of selenium’s effects highlights the importance of maintaining optimal levels rather than administering high-dose supplements indiscriminately. However, the relationship between selenium status and cardiovascular health appears to be complex and may not follow a simple linear “more is better” model [20,21].

A systematic review and meta-analysis by Kuria et al. specifically examined selenium status in the body in relation to cardiovascular disease incidence and mortality. The study found that physiologically high selenium status was associated with a reduced risk of CVD incidence (RR = 0.66; 95% CI: 0.40–1.09) and CVD mortality (RR = 0.69; 95% CI: 0.57–0.84) compared to low selenium status. A 15% decreased risk of CVD incidence was observed per 10 µg increment in blood selenium concentration (RR = 0.85; 95% CI: 0.76–0.94), with the lowest risk at a 30–35 µg increment. For CVD mortality, the dose–response relationship was non-linear, with the lowest estimated risk occurring around a 30–35 μg increment in blood selenium. Nonetheless, the authors highlight that evidence on selenium having protective effects on CVD is still inconclusive, and people should be cautious about potential harmful effects from excessive selenium intake [41].

Detopoulou et al. in the prospective ATTICA study evaluated 278 participants with baseline measurements of selenium-containing selenoproteins and followed them for a mean of 8.74 ± 2.36 years. Total selenium incorporated into selenoproteins was positively associated with 10-year cardiovascular risk, HR 10.02 (95% CI 1.15–92.34) for the third versus second tertile. Notably, participants with relatively high selenium but low GPx3 concentrations also exhibited higher cardiovascular risk, suggesting that selenium status alone may not adequately capture the biological activity of the selenoprotein system [42].

Zinc and selenium are biologically important for redox homeostasis; they may be better considered essential determinants of redox homeostasis rather than conventional antioxidant therapies [21,41].

### 3.5. Mitochondrial-Targeted Antioxidants

Mitochondria are increasingly recognized as key regulators of intracellular signaling and cell fate, with reactive oxygen species (ROS) serving as important mediators of these processes. Consequently, therapeutic approaches that modulate mitochondrial ROS generation may offer a promising strategy to reduce oxidative damage and mitigate the development or progression of cardiovascular disease. [43].

Preclinical studies in aged mice have shown that four weeks of oral MitoQ, a ubiquinol derivative conjugated to a lipophilic triphenylphosphonium cation, accumulates at the inner mitochondrial membrane, where it optimally reduces mtROS, and supplementation with MitoQ completely restored NO-mediated endothelium-dependent dilation, ameliorated mtROS-associated suppression of endothelial function, and reduced age-related aortic stiffness [43,44].

A pilot study in older adults (*n* = 20) demonstrated that MitoQ was well-tolerated, improved endothelial function, and reduced plasma oxidized LDL levels. A larger randomized, placebo-controlled, double-blind trial (NCT04851288) is currently evaluating the effects of 3 months of MitoQ supplementation (20 mg/day) on endothelial function and aortic stiffness in older adults aged ≥ 60 years [45].

Dikalova et al. provided the first direct evidence that mitochondrial oxidative stress plays a causal role in the development of hypertension and endothelial dysfunction. Using cultured endothelial cells exposed to angiotensin II and two experimental models of hypertension (angiotensin II-infused and DOCA-salt mice), the authors demonstrated that mitochondrial superoxide production markedly increased in response to hypertensive stimuli [46].

Treatment with the mitochondria-targeted superoxide scavenger MitoTEMPO significantly reduced mitochondrial and cellular reactive oxygen species (ROS), inhibited NADPH oxidase activation, restored nitric oxide bioavailability, improved endothelial function, and lowered systolic blood pressure by approximately 30 mmHg in both hypertensive models. Furthermore, overexpression of mitochondrial superoxide dismutase (MnSOD/SOD2) reproduced the protective effects of MitoTEMPO, confirming that mitochondrial superoxide is a critical upstream mediator of vascular oxidative stress. These findings highlight the potential of mitochondrial ROS therapies in cardiovascular disease as a strategy to restore vascular redox homeostasis [46].

Liu et al. performed a head-to-head comparison of the mitochondria-targeted antioxidants MitoTEMPO (MT) and Visomitin (SKQ1) under oxidative stress conditions using both in vitro and in vivo models. Oxidative stress was induced in cultured cells by hydrogen peroxide (H_2_O_2_) and menadione, while therapeutic efficacy was further evaluated in a mouse model of renal ischemia–reperfusion injury. Both MT and SKQ1 effectively reduced mitochondrial reactive oxygen species (ROS), restored antioxidant defense gene expression and enzyme activity, attenuated lipid, protein, and DNA oxidative damage, and improved mitochondrial ATP production [47].

However, high concentrations of SKQ1 induced dose-dependent cytotoxicity, whereas MitoTEMPO exhibited a broader therapeutic window. In vivo, MitoTEMPO provided superior protection against ischemia–reperfusion injury, as demonstrated by reduced renal injury biomarkers, improved histopathological outcomes, decreased apoptosis, enhanced mitochondrial function, and greater restoration of antioxidant capacity compared with SKQ1. These findings suggest that although both mitochondria-targeted antioxidants effectively preserve redox homeostasis, MitoTEMPO displays a more favorable efficacy and safety profile, supporting its greater therapeutic potential for oxidative stress–related disorders [47].

### 3.6. Antioxidants’ Current Limitations and Future Directions

#### 3.6.1. The Antioxidant’s Paradox

Overall, the revised evidence on the effectiveness of antioxidants in clinical trials remains controversial. In Table 2, we summarize the main clinical trials of antioxidant interventions in cardiovascular diseases analyzed in this review.

The findings of Khan et al. [48] further emphasize the limitations. Through an umbrella review of 277 randomized controlled trials involving 992,129 participants and 24 nutritional interventions, the authors demonstrated that antioxidant supplements did not significantly reduce all-cause mortality, cardiovascular mortality, myocardial infarction, or stroke in the general population and introduced the concept of the food matrix. This concept explains that antioxidants naturally present in fruits, vegetables, nuts, and extra virgin olive oil interact synergistically with dietary fiber, unsaturated fatty acids, minerals, phytochemicals, and the gut microbiota, collectively influencing their bioavailability, metabolism, and biological activity [48].

However, these findings should not be extrapolated to conclude that oxidative stress is not a valid therapeutic target. Future research should pursue several critical avenues. Although antioxidant therapies have shown favorable effects on oxidative stress biomarkers and endothelial function, the available clinical evidence is heterogeneous and often inconsistent [10,20,49,50]. Well-designed, long-term randomized controlled trials are required to confirm their clinical efficacy and define optimal therapeutic strategies for cardiovascular disease prevention and management [49].

Most of the current evidence is based on preclinical research, including in vitro and animal models, which consistently demonstrate antioxidant-mediated reductions in oxidative stress and vascular injury. However, the translation of these findings into clinical practice remains limited due to the scarcity of high-quality, long-term randomized controlled trials [48,49,50,51].

Translating findings from animal models to human physiology remains challenging because of differences in biological responses, difficulties in selectively delivering therapeutic agents to the vascular wall, and the limited availability of validated surrogate markers for clinical trials. Although antioxidants may provide considerable protection against oxidative stress and its associated pathological effects, their effectiveness can differ substantially according to factors such as dose and origin. These variations highlight the complexity of antioxidant research and the need for careful consideration of experimental and therapeutic conditions [50].

Future research should prioritize the harmonization of experimental methods and the implementation of long-term, multidimensional investigations to clarify the contribution of antioxidants to human health and disease prevention [50].

Currently, lifestyle interventions, including dietary patterns rich in natural antioxidants, caloric restriction, and exercise training, remain the safest and most evidence-based recommendations for preserving vascular health [9].

Recent evidence supports the transition toward precision redox interventions, including mitochondria-targeted antioxidants, Nrf2 activators, and nanotechnology-based delivery systems [10,52].

#### 3.6.2. Personalized Cardiology/Precision Medicine: Biomarker-Guided Therapy

Clinical trial results demonstrating heterogeneous responses to antioxidant interventions have introduced a possible explanation for this wide heterogeneity. The importance of proper patient selection, introducing the idea that the disappointing results of some antioxidants could be explained by poor patient selection.

Precision medicine is increasingly regarded as a promising direction for the future of cardiovascular care, with the potential to provide more individualized and effective strategies for the prevention, diagnosis, and management of cardiovascular diseases than conventional approaches [51].

Several biomarkers have been validated as reliable indicators of oxidative stress. Measuring baseline oxidative stress biomarkers, such as F2-isoprostanes, oxidized LDL (oxLDL), and malondialdehyde, could identify individuals with high oxidative burden who are more likely to benefit from targeted redox-modulating therapies, although prospective biomarker-guided clinical trials are still needed to validate this precision medicine approach and could allow the development of biomarker-guided therapy more precisely and effectively [52,53,54].

oxLDL is not only a marker of oxidative modification of lipoproteins but is also closely associated with vascular dysfunction and cardiovascular disease. Elevated oxLDL levels have been associated with early coronary artery disease and adverse cardiovascular outcomes, supporting its potential value as a marker of an increased oxidative and atherogenic burden [46,47,55].

Oxidized LDL is also an oxidative stress biomarker and has been specifically evaluated as a predictor of cardiovascular outcomes. Zhao et al. demonstrated through a cross-sectional study including 1217 patients with angiography-proven coronary artery disease (CAD) that oxidized LDL is a useful marker for predicting very early coronary artery disease and cardiovascular outcomes, with an adjusted odds ratio of 1.024 (*p* < 0.001) for very early CAD [50].

Furthermore, a very recent multicenter prospective cohort study by Gu et al., including 3733 patients with type 2 diabetes mellitus and coronary heart disease, found that oxidized lipoproteins serve as independent predictors of major adverse cardiovascular events. Through a five years follow up they found that individuals in the highest quartile of oxidized LDL had a significantly elevated 5-year MACE risk (adjusted HR: 2.239; 95% CI: 1.686–2.974; *p* < 0.001) and represents more than a doubling of risk for patients in the highest quartile, independent of traditional risk factors, further demonstrating the prognostic utility of oxidized lipoprotein profiling in clinical risk assessment [52].

Similar results were reported in Gao et al., in a meta-analysis of observational studies with a total of more than 4000 participants, where increased circulating ox-LDL was associated with a pooled effect size of 1.79 (95% CI: 1.56–2.05) for atherosclerotic cardiovascular disease (ASCVD) [53].

These results highlight the potential diagnostic and prognostic utility of oxidative stress biomarkers, reinforce the rationale for incorporating oxidation-specific biomarkers into cardiovascular risk stratification, and support their future integration into biomarker-guided precision cardiology [51,52,53,54].

Nevertheless, it should be mentioned that a major methodological challenge in interpreting clinical evidence on antioxidant interventions is the substantial heterogeneity in oxidative stress biomarkers used across studies. Different trials have employed distinct biomarkers, including malondialdehyde (MDA), oxidized LDL (oxLDL), F2-isoprostanes, 8-hydroxy-2′-deoxyguanosine (8-OHdG), protein carbonyls, and advanced oxidation protein products (AOPP), each reflecting different aspects of oxidative damage and measured using different methodologies. This variation in biomarker selection and methodology not only limits direct comparability but also may contribute to the inconsistent findings across antioxidant trials [3,4,10,56]. Thereby oxidative stress is not a single measurable biological entity. Changes in different biomarkers should not be interpreted as equivalent evidence of an overall reduction in oxidative stress. Despite these limitations, oxidative stress biomarkers have demonstrated significant prognostic and risk-stratification value. Future clinical studies would benefit from standardized, multidimensional biomarker panels that integrate oxidative damage, redox status, and endogenous antioxidant defenses rather than relying on a single biomarker [10,50,55].

Although prospective trials evaluating biomarker-guided antioxidant therapy are still lacking, oxidation-specific biomarkers have demonstrated significant prognostic and risk-stratification value, supporting their potential use for identifying patients with a high oxidative stress phenotype who may be candidates for future personalized redox-modulating interventions [10,52,53].

The potential clinical application of antioxidant interventions should be considered within the context of the underlying cardiovascular risk profile, nutritional status, concomitant pharmacotherapy, and the specific antioxidant compound. Current evidence does not support a generalized recommendation for antioxidant supplementation as a strategy for primary or secondary cardiovascular prevention [57,58]. In generally healthy individuals with an adequate dietary intake of antioxidant-rich foods, routine supplementation with isolated antioxidant compounds is not supported by sufficient evidence to justify replacing established dietary and lifestyle measures. In patients with stable atherosclerotic disease, antioxidants should likewise not be considered substitutes for evidence-based therapies such as lipid-lowering, antihypertensive, antiplatelet, or glucose-lowering treatment when indicated. Their potential role is better regarded as adjunctive and remains investigational, particularly because clinical trials have generally assessed surrogate biomarkers rather than cardiovascular events. In patients receiving secondary prevention pharmacotherapy, any potential antioxidant intervention should therefore be evaluated according to the specific compound, dose, nutritional status, and concomitant medications rather than applied as a class-wide strategy [56,57,58].

The concept of “proper patient selection” has evolved into a broader precision medicine framework. Rather than administering antioxidant therapies indiscriminately, current evidence supports identifying patients with distinct biological phenotypes using circulating biomarkers, imaging modalities, and molecular profiling. This biomarker-driven approach aims to identify individuals with a predominant oxidative stress phenotype who may derive the greatest benefit from targeted redox-modulating therapies [52,53,59].

#### 3.6.3. Possible Antioxidant Interactions

Beyond efficacy signals, antioxidants should not be considered a pharmacologically homogeneous class or risk-free therapies. Although the safety profile is favorable in most of them, there are specific interactions that should be considered, especially in cardiovascular patients already receiving standard medication [8].

Resveratrol at high doses inhibits cytochrome P450 enzymes, particularly CYP3A4, increasing plasma concentrations of drugs metabolized by CYP3A4. Preclinical evidence indicates that resveratrol can alter warfarin pharmacokinetics and enhance anticoagulant activity; however, direct clinical evidence in warfarin-treated patients remains limited [60].

High-dose omega-3 fatty acids (>3 g/day) exhibit antiplatelet activity and may increase bleeding risk when combined with anticoagulants or antiplatelet agents. Although the evidence is not homogeneous, in a systematic review and meta-analysis, Javaid et al. show that there is not a generalized increase in bleeding risk. However, high-dose purified EPA has been associated with a modest, dose-dependent increase in bleeding, suggesting that the safety profile may vary by formulation and dose [61].

High-dose curcumin (≥1 g/day) inhibits CYP3A4 and may increase plasma levels of drugs metabolized by CYP3A4, such as statins and others; monitoring is recommended, an interaction that appears dose-dependent. Jiang et al. investigated in rats and with rat liver microsomes the drug–drug interaction between curcumin and amlodipine, a commonly used antihypertensive, and curcumin significantly increased systemic amlodipine exposure, with Cmax rising from 17.80 to 26.19 μg/L and AUC from 238.68 to 507.27 μg·h/L [62,63].

Regarding selenium, although some recent reviews have mentioned potential bleeding interactions between selenium supplementation and antithrombotic therapy, clinically robust evidence supporting a clinically significant interaction remains limited [64]. Yet the most recent update from the NIH Office of Dietary Supplements (ODS) identifies a cisplatin and selenium interaction and mentions that cisplatin can lower selenium levels, but no clear clinical significance is established. It also highlights that some small studies have suggested that selenium could reduce cisplatin toxicity, although the evidence is insufficient [65,66].

Zinc interactions with cardiovascular drugs have no strong evidence, but remarkably, oral zinc supplementation may reduce the absorption of antibiotics such as ciprofloxacin and doxycycline, whereas diuretics and angiotensin-receptor blockers may contribute to zinc deficiency [67].

CoQ10 is structurally related to vitamin K and may attenuate the anticoagulant effect of warfarin; previous case reports have suggested a possible relation with reduced INR. Although the clinical evidence remains inconsistent, INR monitoring may be prudent when CoQ10 supplementation is initiated in patients receiving warfarin [64].

Collectively, these interactions underscore that antioxidant supplementation in cardiovascular patients should not be regarded as inert and requires pharmacovigilance [10,62].

### 3.7. Senolytic Therapies: An Emerging Strategy for Vascular Aging

Oxidative stress and cellular senescence are mechanistically interconnected but biologically distinct processes; oxidative stress can promote DNA damage, mitochondrial dysfunction, and activation of senescence pathways (p53/p21 and p16^INK4a^/Rb signaling) [5,14], whereas established senescent cells sustain a pro-oxidant and pro-inflammatory tissue environment through the senescence-associated secretory phenotype (SASP) [11,14,15]. This bidirectional relationship generates a self-perpetuating cycle in the vasculature, in which oxidative damage drives senescence and senescence amplifies local oxidative and inflammatory burden. Considering senolytic strategies, agents that selectively clear senescent cells could be very useful as a complementary and potentially non-redundant therapeutic strategy alongside antioxidant approaches in the context of vascular aging [5,11,14].

Senolytics are a class of pharmacological agents that selectively induce apoptosis in senescent cells by targeting senescent cell anti-apoptotic pathways (SCAPs), including BCL-2/BCL-XL and PI3K/AKT signaling, on which senescent cells become dependent for survival despite accumulated damage, while sparing viable, non-senescent cells [1,5]. Broad-spectrum combination agents include dasatinib plus quercetin [1,5].

The most extensively studied senolytic combination in the cardiovascular context is dasatinib plus quercetin (D + Q). Dasatinib, a tyrosine kinase inhibitor, and quercetin, a natural flavonoid with antioxidative and anti-inflammatory properties, have distinct cell-type specificities: dasatinib is active in preadipocytes and quercetin in endothelial cells, and they are more effective when administered together [1,7,12].

Triana-Martínez et al. [68] identified cardiac glycosides (CGs), including digoxin, as potent senolytic agents through inhibition of the Na^+^/K^+^-ATPase, and demonstrated preferential killing of several types of senescent human cells compared with proliferating or quiescent cells. This mechanism disrupts intracellular Na^+^/K^+^ homeostasis and promotes plasma-membrane depolarization and intracellular acidification. Because senescent cells exhibit a partially depolarized membrane potential and elevated intracellular H^+^ concentrations, they are particularly vulnerable to further disruption induced by cardiac glycosides, ultimately leading predominantly to apoptotic cell death. Importantly, it should be noted that clinical evidence for glycosides in this context is still limited [68].

Roos et al. provided preclinical evidence investigating the effects of long-term Dasatinib (5 mg/kg) + Quercetin (50 mg/kg) biweekly for 8 months in chronologically aged C57BL/6J or hypercholesterolemic mice with established disease. Senolytic treatment significantly reduced senescent cell markers (TAF+ cells) in the medial layer of the aorta, but not in intimal atherosclerotic plaques. Senolytics also improved NO bioavailability/signaling, with the consequent improvement in vasomotor function; Aortic medial calcification also tended to decrease. This was the first study to demonstrate that chronic clearance of senescent cells improves established vascular phenotypes associated with aging [69].

More recently, Mury et al. [70] developed the Q-CABG trial, a prospective, multicenter, randomized, double-blind, placebo-controlled clinical trial enrolling 111 patients with recent acute coronary syndrome scheduled for elective coronary artery bypass grafting (CABG). Patients received Quercetin 500 mg twice daily (1000 mg/day) vs. placebo, initiated 2 days before CABG and continued until hospital discharge. The results showed that Quercetin produced a trend toward lower CRP at hospital discharge, although this did not reach conventional statistical significance (*p* = 0.073), also improved acetylcholine-induced endothelial relaxation (*p* = 0.049), notably this effect was sex dependent, with a significant effect in men (*p* = 0.043) but not women (*p* = 0.852); and vascular transcriptomics indicated reduced senescence/inflammaging signatures in men. Clinically, quercetin reduced the incidence of post-operative atrial fibrillation compared to placebo (4% vs. 18%, *p* = 0.033). These findings demonstrate that in male patients with coronary artery disease (CAD), short-term quercetin administration appeared to mitigate vascular senescence and improve both inflammatory markers and vascular function. However, comparable effects were not demonstrated in female patients, in whom quercetin treatment was associated with a potential increase in pro-inflammatory responses [70].

Lifestyle interventions, particularly exercise, have also been shown to act as a senolytic, preventing the accumulation of senescent cells accompanied by reduced oxidative stress and inflammation [6]. Larger trials are needed to confirm safety and efficacy, and future research should investigate whether combining senolytics with targeted antioxidant strategies yields additive or synergistic benefits in delaying cardiovascular aging [5,9]. Future research should investigate whether combining senolytics with targeted antioxidant strategies yields additive or synergistic benefits in delaying cardiovascular aging [9].

## 4. Conclusions

Oxidative stress is a central driver of cardiovascular disease (CVD), contributing to endothelial dysfunction, chronic inflammation, mitochondrial dysfunction, vascular remodeling, myocardial injury, and the progression of atherosclerosis, hypertension, heart failure, and arrhythmias.

Natural antioxidants, including resveratrol, coenzyme Q10, vitamins C and E, omega-3 fatty acids, and other polyphenols, demonstrate promising antioxidant, anti-inflammatory, and endothelial-protective effects in experimental studies. However, clinical efficacy remains inconsistent due to limitations in bioavailability, pharmacokinetics, dosing strategies, and variability among clinical trials. The effectiveness of an antioxidant depends on the pathophysiological context, the dose, the bioavailability, and the type of disease

Lifestyle interventions, especially adherence to antioxidant-rich dietary patterns such as the Mediterranean diet, remain essential strategies for reducing oxidative stress and cardiovascular risk.

Combining lifestyle modification and targeted antioxidant strategies may provide the greatest potential for oxidative stress-associated cardiovascular diseases.

There is a need for precision medicine approaches and well-designed randomized clinical trials to identify patients most likely to benefit from antioxidant interventions.

## Figures and Tables

**Table 1:** Endogenous antioxidants and exogenous antioxidants/redox-modulating compounds based on references [16,19,20,21,22].

Endogenous Antioxidants	Exogenous Antioxidants	Type of Compound	Solubility	Redox-Related Pathways and Molecular Mechanisms
Superoxide dismutase (SOD1, SOD2, SOD3)	Resveratrol	Polyphenol (Stilbenoid)	Lipophilic	Redox modulation; SIRT1 activation; Nrf2 pathway activation; inhibition of NF-κB signaling.
Catalase (CAT)	Quercetin	Flavonoid	Lipophilic	Redox modulation; Nrf2 activation; inhibition of oxidative and inflammatory signaling.
Glutathione peroxidases (GPx)	β-Carotene	Carotenoid	Lipophilic	Singlet-oxygen quenching; peroxyl-radical scavenging; provitamin A activity.
Peroxiredoxins (Prx)	Lycopene	Carotenoid	Lipophilic	Singlet-oxygen quenching; peroxyl-radical scavenging.
Thioredoxin (Trx)/Thioredoxin reductase	Vitamin E (α-Tocopherol)	Lipid-soluble vitamin	Lipophilic	Chain-breaking inhibition of lipid peroxidation; inhibition of lipid autoxidation; interaction with vitamin C-dependent antioxidant recycling.
Glutathione (GSH)	Curcumin	Polyphenol (Diarylheptanoid)	Lipophilic	Nrf2 activation; modulation of NF-κB and MAPK signaling; downregulation of COX-2/iNOS.
Coenzyme Q10 (Ubiquinol)	Epigallocatechin gallate (EGCG)	Polyphenol	Lipophilic	ROS scavenging; mitochondrial electron transport; regeneration of vitamin E
Melatonin	Selenium	Trace mineral	Mineral	Component of selenoproteins (GPx, TrxR); cofactor for antioxidant enzymes
Bilirubin	Zinc	Trace mineral	Mineral	Cofactor for SOD; enzyme stabilizer; metallothionein induction; NADPH oxidase inhibition

**Table 2:** Summary Evidence on Antioxidant Interventions in Cardiovascular Health.

Antioxidant/Intervention	Year	Study Design/Model	Sample	Intervention/Exposure	Main Outcome(s)	Limitations
Mediterranean Diet CORDIOPREV [24]	2018	RCT	n = 805, coronary heart disease patients	Mediterranean diet + ≥40 mL/day olive oil, 5 years	Slower CIMT progression compared with low-fat diet	Antioxidant-specific effects cannot be isolated
Mediterranean Diet PREDIMED [25]	2026	RCT	n = 1180 (653 incident CVD cases + subcohort)	Mediterranean diet; urinary polyphenol signature	Cardiovascular outcomes	Multicomponent intervention
Resveratrol [28]	2017	RCT, DB, PC, crossover	n = 30 older adults with IGT	Resveratrol 2–3 g/day, 6 weeks per treatment period	Improved RHI (*p* = 0.002); ↑ mitochondrial number; no effect on glucose metabolism	Small sample
Resveratrol [27]	2018	RCT, DB, PC, crossover	n = 24, hypertensive adults 45–65	Acute trans-resveratrol supplementation	FMD increased in women (4.2 → 7.1%, *p* = 0.026) but not men; no BP change	Small sample; acute intervention
Carotenoids [32]	2022	Prospective cohort (NHANES)	n = 3107 T2DM	Baseline serum α-carotene, β-carotene, β-cryptoxanthin, lycopene, lutein/zeaxanthin	↑ β-carotene associated with ↑ CVD mortality (HR 2.47; 95% CI 1.62–3.76; P-trend = 0.002)	Prospective observational evidence
Omega-3 VITAL [34]	2019	RCT, DB, PC	n = 25,871 primary preventions	1 g/day omega-3; median 5.3 years	MACE: HR 0.92 (95% CI 0.80–1.06; *p* = 0.24); No significant effect on total stroke, CV death, or CABG	Large human RCT
Omega-3 STRENGTH [35]	2020	RCT, DB, PC	>13,000 high CV risk	4 g/day EPA + DHA vs. corn oil	No MACE reduction; terminated for futility; ↑ atrial fibrillation	Large human RCT
Curcumin [37]	2017	RCT, DB, PC	n = 39 healthy adults, 45–74 years	Longvida^®^ curcumin 2000 mg/day, 12 weeks	↑ FMD (~6.0 → 7.7%, *p* < 0.01); ↑ NO bioavailability; no change in arterial stiffness	Human intervention; small sample
Curcumin [38]	2023	RCT, DB, PC	n = 64, T2DM + mild-moderate CAD	Nano-curcumin 80 mg/day, 90 days	Reduced hs-CRP (*p* < 0.001) and Lp(a) (*p* = 0.043) vs. placebo; percentage changes also significant	Human RCT; small sample
Zinc [40]	2020	RCT, DB, PC	60 patients with T2DM and CHD	250 mg/day magnesium oxide + 150 mg/day zinc sulfate (30 mg elemental zinc/day) for 12 weeks	Co-supplementation significantly reduced CRP (β = −0.85 mg/L; *p* < 0.001) and increased total antioxidant capacity (β = 43.44 mmol/L; *p* = 0.03) and total nitrite (β = 5.13 μmol/L; *p* = 0.003).	Zinc-specific effects cannot be isolated. Small sample and short intervention period.
Selenium [42]	2023	Prospective observational cohort	n = 278; mean follow-up 8.74 ± 2.36 years	Baseline Se in selenoproteins and GPx3	Total Se in selenoproteins: HR 10.02 (3rd vs. 2nd tertile; 95% CI 1.15–92.34) for 10-year CVD risk	Prospective observational evidence
MitoQ [45]	2018	Human intervention study	n = 20 older adults	Chronic MitoQ supplementation	Improved vascular function and reduced plasma oxidized LDL	Human intervention; small sample
MitoTEMPO/SKQ1 [11]	2024	In vitro + in vivo (renal I/R model)	Cell and mouse models	MitoTEMPO vs. SKQ1 under oxidative stress	Both ↓ mtROS; MT broader therapeutic window; SKQ1 dose-dependent cytotoxicity	Preclinical; in vitro/in vivo

Symbol explanations: (↓) decrease; (↑) increase; arrow (→) between numerical values indicates the reported change from one value to another. Arrows are descriptive and do not independently indicate statistical significance.

## Data Availability

No new data were created or analyzed in this study. Data sharing is not applicable to this article.

## Supplementary Materials

The following supporting information can be downloaded at: https://www.mdpi.com/article/10.3390/antiox15091152/s1. Table S1: Methodological quality, risk of bias, and evidence characteristics of the studies included in the review.

## Abbreviations

The following abbreviations are used in this manuscript:

8-OHdG	8-Hydroxy-2′-deoxyguanosine
ATP	Adenosine Triphosphate
AngII	Angiotensin II
BP	Blood pressure
CAD	Coronary Artery Disease
CAT	Catalase
CHD	Coronary Heart Disease
CIMT	Carotid Intima-Media Thickness
CVD	Cardiovascular Disease
DNA	Deoxyribonucleic Acid
eNOS	Endothelial Nitric Oxide Synthase
GPx	Glutathione Peroxidase
GSH	Reduced Glutathione
GSSG	Oxidized Glutathione
H_2_O_2_	Hydrogen Peroxide
HF	Heart Failure
IL	Interleukin
LDL	Low-Density Lipoprotein
MACE	Major Adverse Cardiovascular Events
MDA	Malondialdehyde
MPO	Myeloperoxidase
mtROS	Mitochondrial Reactive Oxygen Species
NF-κB	Nuclear Factor Kappa B
NO	Nitric Oxide
NOX	NADPH Oxidase
Nrf2	Nuclear Factor Erythroid 2-Related Factor 2
OxLDL	Oxidized Low-Density Lipoprotein
OxPL-apoB	Oxidized Phospholipids on Apolipoprotein B
RCT	Randomized Controlled Trial
RNS	Reactive Nitrogen Species
ROS	Reactive Oxygen Species
SASP	Senescence-Associated Secretory Phenotype
SOD	Superoxide Dismutase
TNF-α	Tumor necrosis factor alpha
VCAM-1	Vascular cell adhesion molecule-1
VSMCs	Vascular Smooth Muscle Cells
