# Supplementary material for: Oxidative Stress in Vascular Aging: Therapeutic Potential of the Antioxidant Paradox. A State-of-the-Art Review
Source: Antioxidants (Basel). 2026 Sep 10;15(9):1152. doi: 10.3390/antiox15091152 (PMC13603535; doi:10.3390/antiox15091152)
Supplement: Supplementary file 1 [file antioxidants-15-01152-s001.zip › antioxidants-4490462-supplementary.pdf]

Supplementary Table S1. Methodological quality, risk of bias, and evidence characteristics of the studies included in the review.

A. Systematic reviews, meta-analyses, and umbrella reviews quality assessment (AMSTAR 2)

Antioxidant/intervention	Reference	Study design	Evidence base	Main outcome	Assessment Result	Key limitation
Oxidative-stress interventions in CVD	Jin et al. 2024 [10]	Systematic review	Clinical and experimental evidence	Management of oxidative-stress-associated CVD	Moderate (No protocol registration)	Heterogeneity of interventions and outcomes
Zinc	Nazari et al., 2023 [39]	Systematic review + dose-response meta-analysis of RCTs	75 RCTs	Cardiometabolic, inflammatory and oxidative-stress risk factors	High	Outcomes predominantly surrogate; heterogeneous populations/doses
Selenium	Kuria et al., 2021 [41]	Systematic review + meta-analysis	43 RCTs / selenium-status evidence	Selenium status and CVD incidence/mortality	Moderate	Observational associations and supplementation evidence are not equivalent
Antioxidant/nutritional interventions	Khan et al., 2019 [48]	Umbrella review + evidence map	277 RCTs; 992,129 participants; 24 interventions	Mortality, MI, stroke and CVD outcomes	High	Nutritional interventions heterogeneous; food matrix differs from isolated supplements

B. Randomized controlled trials quality RoB 2 assessment

Antioxidant	Reference	Design	Sample	Intervention	Comparator	Follow-up	Main outcome	Main finding	Assessment Result	Key limitation

Mediterranean diet	Rivas-Garcia et al. / CORDIOPREV, 2024 [24]	RCT	**n=805**	Mediterranean diet + ≥40 mL/day olive oil	Low-fat diet	5 years	CIMT progression	Lower CIMT progression	Low RoB	Multicomponent intervention
Resveratrol	Pollack et al., 2017 [27]	RCT, DB, PC, Crossover	**n=30**	2–3 g/day	Placebo	6 weeks/treatment period	RHI, mitochondrial outcomes	Improved RHI and mitochondrial number	Some concerns	Small sample; short duration
Resveratrol	Marques et al., 2018 [28]	RCT, DB, PC, Crossover	**n=24**	Acute trans-resveratrol	Placebo	Acute	FMD, BP	FMD improved in women, no BP effect	Some concerns	Very small sample; acute intervention
Omega-3	Manson et al. / VITAL, 2019 [34]	RCT, DB, PC	**n=25,871**	1 g/day marine n-3	Placebo	Median 5.3 y	MACE	No significants	Low RoB	dose/formulation specific
Omega-3	Nicholls et al. / STRENGTH, 2020 [35]	RCT, DB, PC	n= >13,000	4 g/day EPA+DHA	Corn oil	Terminated early	MACE	No CV benefit	Low RoB	Early termination for futility
Curcumin	Santos-Parker et al., 2017 [37]	RCT	n=39	Longvida® curcumin 2 g/day	Placebo	12 weeks	FMD, NO bioavailability	Improved FMD and NO bioavailability	Some concerns	Small sample; surrogate endpoints
Curcumin	Dastani et al., 2023 [38]	RCT, DB, PC	**n=64**	Nano-curcumin 80 mg/day	Placebo	90 days	hs-CRP, Lp(a)	Reduced hs-CRP and Lp(a)	Some concerns	Small sample; surrogate outcomes

Zinc + magnesium	Hamedifard et al., 2020 [40]	RCT, DB, PC	**n=60**	MgO 250 mg/day + zinc sulfate 150 mg/day	Placebo	12 weeks	TAC, nitrite, CRP/metabolic outcomes	Improved TAC and nitrite; reduced CRP	Some concerns	Combined intervention; zinc-specific effect cannot be isolated
Mito Q	Rossman et al., 2018 [45]	Pilot RCT	n=20	Oral MitoQ, 20 mg/day	Placebo	Short term	Brachial artery flow-mediated dilation	↑ FMD~BA~ vs. placebo, ↓ oxidized LDL	High RoB	Very small sample, surrogate outcomes

C. Observational / prospective cohort studies quality assessment with Newcastle-Ottawa Scale

**Antioxidant**	**Reference**	**Design**	**Sample**	**Exposure**	**Follow-up**	**Outcome**	**Main finding**	**Assessment result**	**Key limitation**
Carotenoids	Qiu et al., 2022 [32]	Prospective cohort (NHANES).	T2DM; **n=3,107**	Serum α-carotene, β-carotene, β-cryptoxanthin, lycopene, lutein/zeaxanthin	~14 years	CV mortality	Higher β-carotene associated with higher CV mortality; HR 2.47 (95% CI 1.62–3.76)	High quality	Residual confounding; association ≠ causation
Selenium	Detopoulou et al. / ATTICA, 2023 [42]	Prospective cohort	**n=278**	Selenium in selenoproteins / GPx3	8.74 ± 2.36 years	10-year CV risk	Higher Se in selenoproteins associated with ↑ 10-year CV risk: HR 10.02 (95% CI 1.15–92.34)	High quality	Small cohort; wide CI; association ≠ supplementation benefit

D. Preclinical studies quality assessment (SYRCLE)

**Antioxidant**	**Reference**	**Study design**	**Intervention**	**Main outcome**	**Key finding**	**Assesment**	**Key translational limitation**

Hexahydrocurcumin	Panthiya et al., 2023 [36]	In vitro (rat VSMCs)	HHC, 20 μM	ROS, NF-κB, inflammatory/remodeling markers, VSMC migration/proliferation	Reduced oxidative stress and VSMC remodeling	Low quality	In vitro model;
MitoQ	Gioscia-Ryan et al., 2014 [43]	In vivo (aged mice)	Oral MitoQ, 4 weeks	Endothelium-dependent vasodilation	Restored NO-mediated endothelial dilation; reduced mtROS	Moderate quality	Animal model; no clinical translation
MitoQ	Gioscia-Ryan et al., 2018 [44]	In vivo (old mice)	MitoQ	Aortic stiffness	Attenuated age-related aortic stiffening	Moderate quality	Animal model
MitoTEMPO	Dikalova et al., 2010 [46]	In vitro + in vivo (hypertensive mice)	MitoTEMPO	mtROS, NO bioavailability, endothelial function, BP	MitoTEMPO ↓ mitochondrial superoxide and BP (~30 mmHg) .	Moderate quality	Mechanistic and animal evidence; no clinical validation
MitoTEMPO vs. SKQ1	Liu et al., 2024 [11]	In vitro + in vivo (renal I/R)	MitoTEMPO vs. SKQ1	mtROS, oxidative damage, mitochondrial function	Both agents reduced oxidative injury;	Moderate quality	No human evidence
